# Auditory perception of biodiversity by human listeners

**DOI:** 10.3389/fpsyg.2025.1552329

**Published:** 2025-07-08

**Authors:** Elie Grinfeder, Jérôme Sueur, Richard McWalter, Frédéric Apoux, Christian Lorenzi

**Affiliations:** ^1^Laboratoire des Systèmes Perceptifs (LSP), CNRS, Ecole normale supérieure, Université Paris Sciences & Lettres (PSL University), Paris, France; ^2^Centre d'Ecologie et des Sciences de la conservation (CESCO), Muséum national d'Histoire naturelle, Centre national de la Recherche Scientifique, Sorbonne Université, Paris, France

**Keywords:** human auditory ecology, soundscape ecology, biodiversity perception, bird chorus, noise pollution

## Abstract

**Introduction:**

This study explored human auditory capacity to evaluate the number of biological sound sources in natural soundscapes.

**Methods:**

This was achieved by measuring the ability of human participants to judge the number of birds when listening to soundscapes generated by an engineering algorithm that controlled for bird abundance, species richness, level disparities between songs, bird behavior and background noise.

**Results and discussion:**

Although often inaccurate, numerosity judgments were generally affected by the number of birds, demonstrating sub-optimal sensitivity to biodiversity in humans. Numerosity judgments were robust to low-intensity background sounds, and higher when between-species acoustic disparities were introduced, suggesting that grouping mechanisms contribute to biodiversity perception.

## 1 Introduction

Natural environments, that is environments marginally affected by human activity (e.g., protected natural areas, national parks), generate soundscapes resulting from the combination of different sounds of geophysical (wind, rain, streams), biological (animal vocalizations) or anthropological (human vocalization, machine noise) origin (Pijanowski et al., 2011). These categories of collective sounds were named geophony, biophony, and anthropophony respectively (Krause, 1987). In an attempt to specify further the soundscape concept, its proximal form was recently defined as the collection of propagated geophysical and biological sounds that may be recorded at a specific point in space (e.g., at the ear receptor of a biological organism). The proximal soundscape was distinguished from the perceptual soundscape, defined as the individual and therefore subjective interpretation of the proximal soundscape resulting from the operation of auditory, cognitive and emotional processes in the ear and brain of the listening organism (Grinfeder et al., 2022a). Human auditory ecology aims at studying how human listeners build perceptual soundscapes from proximal ones in the case of natural environments (Lorenzi et al., 2023). An important goal of human auditory ecology is to understand the extent to which and how human listeners perceive biodiversity in a given environment through their ear and brain. Here, biodiversity was studied under two of its main descriptive components, that is species abundance (number of individuals attributed to a single species), and species richness (number of species). This endeavor is motivated by the results of questionnaire-based surveys revealing that the amount of wellbeing felt by humans visiting a green space (e.g., a park) is systematically modulated by the number of bird songs occurring in the area (Ferraro et al., 2020; Fuller et al., 2007). These preliminary findings suggest that the amount of biodiversity may correspond to an important feature of auditory scenes for human listeners, that is not only perceived as such but also taken into account to assess the quality of the close environment. This is in line with recent work suggesting that pervasiveness of human-induced noise pollution in natural areas has an impact on the human capacity to perceive soundscapes, assess their biodiversity and benefit from them (Buxton et al., 2017, 2021; Dominoni et al., 2020).

The goal of the present study was to identify the ecological and auditory factors that may influence auditory perception of biodiversity in natural environments for human listeners. A dedicated soundscape engineering algorithm named “Evascape” (Grinfeder et al., 2024) was used to generate proximal soundscapes derived from a functional and ecologically-valid model of natural-soundscape generation. This soundscape assembler was built upon a critical review of ecological and acoustic factors involved in the content and organization of perceptual soundscapes (Grinfeder et al., 2022a). Evascape aims at reproducing realistic proximal soundscapes that may be recorded in a given habitat (here, a temperate, cold coniferous forest in France). To achieve this goal, Evascape assembles recordings from birds of the same species or different species with congruent acoustic backgrounds produced by biotic, abiotic and anthropogenic sources. In addition, Evascape offers the possibility to simulate aspects of bird singing behavior such as a temporal-avoidance strategy, mimicking the active partitioning of acoustic space in bird communities. Finally, Evascape simulates the specific characteristics of sound propagation in the habitat under study. Consequently, the Evascape assembler opens the possibility to study two factors expected to influence the auditory organization of perceived natural scenes for human listeners:

1. If perceptually salient enough for the human auditory system, acoustic disparities between bird vocalizations should influence the simultaneous and sequential grouping of songs into distinct auditory streams by human listeners, as shown previously by psychoacoustic studies conducted with artificial, musical and speech sounds (Bregman, 1990; for a review see Moore and Gockel, 2012). Disparities within- and between-species correspond to differences in spectro-temporal acoustic patterns and loudness (see Catchpole and Slater, 2003). These disparities, which are a facet of biodiversity, result distally from sexual and natural selection and proximally from phenotypic factors such as the size and shape of the syrinx or from acquired traits which are transmitted across populations as a form a local culture (Aplin, 2019). Following the acoustic niche hypothesis (ANH) formulated by Krause (1987), between-species differences would limit competition for the acoustic space, each species occupying a specific acoustic niche. This acoustic avoidance would then lead to acoustic space partitioning. Loudness disparities result from differences in song level across bird species (Catchpole and Slater, 2003), and distance between individual birds and receiver. In Evascape, the effects of distance on sound propagation were simulated by applying a frequency-dependent attenuation filter derived from acoustic measures performed *in situ*. In relation with evolutionary constraints, such as species behavioral isolation, bird song interspecific differences are expected to be greater than intraspecific differences (Catchpole and Slater, 2003). As a consequence, auditory segregation of bird songs by human listeners should be more effective – and thus, the perceived number of birds should be higher – for a community chorus composed of birds from different species compared to a population chorus composed of birds from the same species. In the same vein, loudness disparities between species should enhance auditory segregation and perceived number of birds.
2. Spectro-temporal overlap of biotic, abiotic and anthropogenic sound sources is expected to produce both energetic, modulation and informational masking effects, depending on the spectro-temporal structure of each sound source and their similarities, as demonstrated previously for human listeners with artificial and speech sounds, mainly in the case of urban settings (Durlach et al., 2003; Dubbelboer and Houtgast, 2008). All three forms of masking result from the superimposition of bird songs with biophony, geophony (e.g., wind or rain), ambient sound and anthropophony (e.g., aircraft noise). In the first case, masking arises simultaneously from the overlap of bird songs, and from other acoustics competitors such as sounds produced by insects, anurans and mammals. This overlap should decrease signal-to-noise ratio in the audio and modulation domains to the receiver ear. It should also cause distraction and confusions, degrading further auditory processing of each bird song. On the other hand, song overlapping and consequent masking effects may be reduced by interactive processes regulating bird singing behavior (Cody and Brown, 1969; Ficken et al., 1974; Popp et al., 1985; Brumm, 2006; Planqué and Slabbekoorn, 2008; Suzuki et al., 2012). Indeed, in certain situations, birds may adopt temporal-avoidance strategies that minimize spectro-temporal overlap between their own song and that of other (competing) birds, inserting intentionally their own vocalizations into the spectro-temporal valleys of other bird songs (Brumm, 2006). Local reduction in signal-to-noise ratio should offer human listeners opportunities to glimpse segments of bird song within natural scenes, as demonstrated previously for active speech communication in humans (Cooke and Lu, 2010; Füllgrabe et al., 2006). In addition, it is expected that active changes in temporal patterns of bird songs – if perceptually detectable by humans – should produce temporal cues (i.e., differences in rhythmic patterns) susceptible to reduce modulation-masking effects. A temporal-avoidance strategy was simulated by Evascape to assess the capacity of human listeners to benefit from the expected release from energetic and modulation masking caused by active song interlacing.

Here, the auditory ability of human listeners to assess biodiversity abundance and richness in complex acoustic scenes and the contribution of basic streaming and glimpsing auditory mechanisms were studied using a numerosity judgment task. This judgment was made upon a database of engineered soundscapes generated by Evascape to reproduce ecologically valid acoustic recordings encountered in a cold, temperate, coniferous forest in France including an important level of anthropophony due to aircraft traffic. A single-judgment method was used to minimize response biases typically associated with numerosity tasks (Warren, 1970; Krueger, 1982). All psychoacoustic experiments were conducted online as single-judgment methods typically require a large number of participants.

## 2 Material and methods

### 2.1 Habitat under study: Risoux forest, France

**Figure 1:**
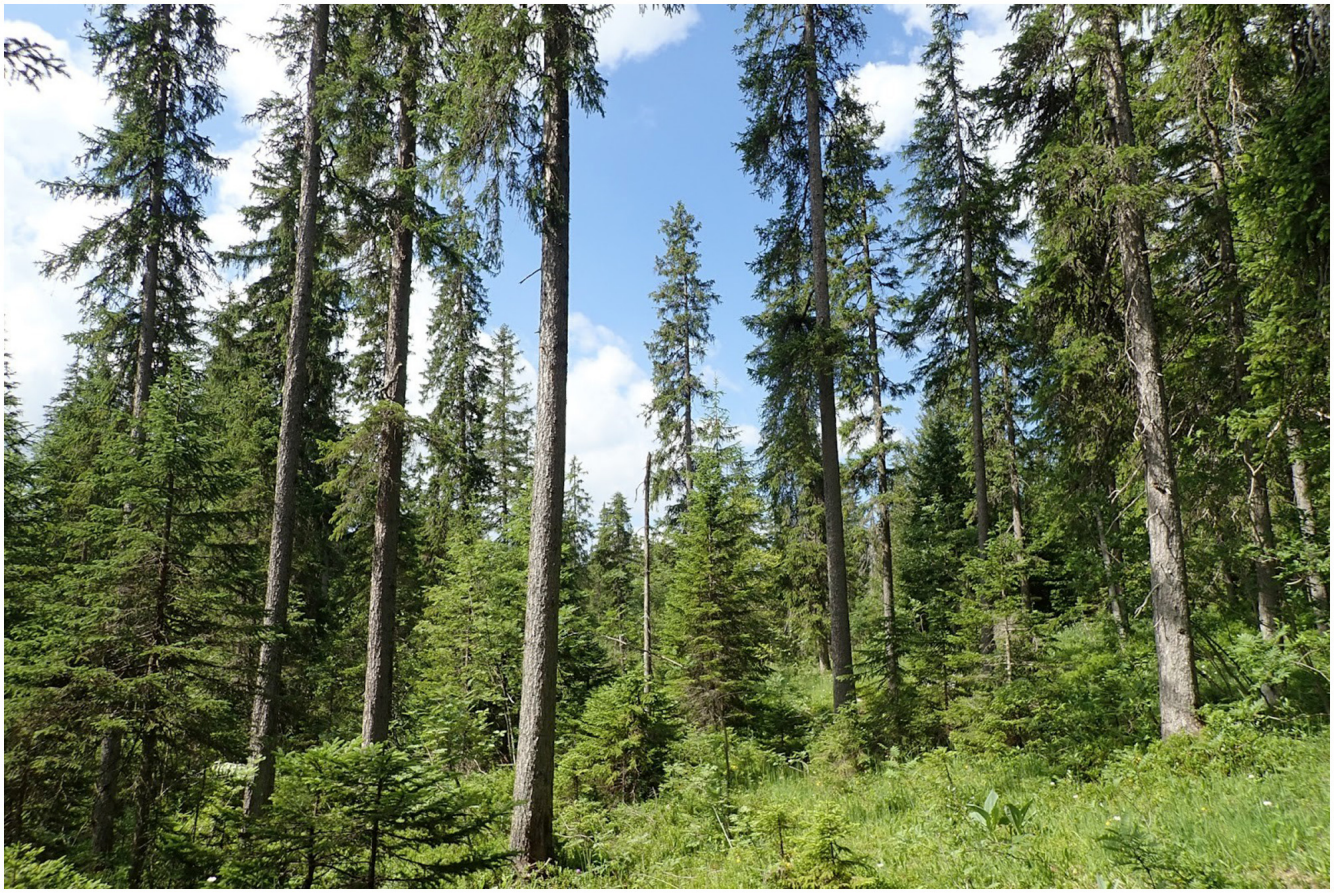
The Risoux forest in the summer. In its inner part, the forest is dominated by the European spruce tree (*Picea abies*) (Credit: J. Sueur).

The Risoux forest is a protected European cold forest located in the East of France, in the Haut-Jura mountains. Its medium altitude (1,230 m a. s. l.) and anticlinal shape result in a cold climate inducing a short vegetation period between April and October (Figure 1). A 15-year long recording procedure was initiated in July 2018 based on the deployment of four autonomous recorders regularly dispatched along the forest recording 1 min every 15 min. A preliminary study based on a machine learning technique unveiled the temporal acoustic patterns of the Risoux-forest distal soundscape, and the pervasiveness of aircraft noise (Grinfeder et al., 2022b).

### 2.2 Assemblage of proximal soundscapes: the Evascape software

**Figure 2:**
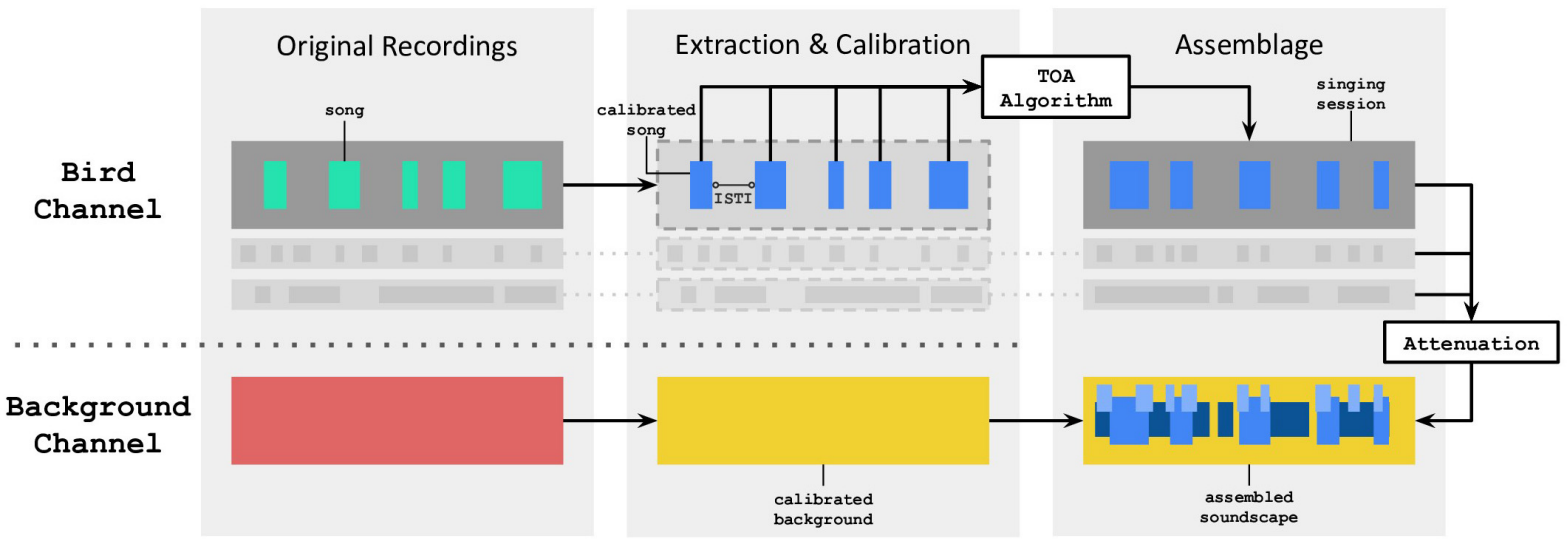
Signal processing flow of the Evascape assembler, mainly based on two channels, one dedicated to bird vocalizations, the other one to background sounds. ISTI stands for Inter-Song Time Interval. TOA stands for Temporal Overlap Avoidance.

Acoustic stimuli were crafted based on the Evascape soundscape assembler which can produce ecologically-valid bird choruses of the Risoux cold forest (Grinfeder et al., 2024). The assembled soundscapes are obtained by combining with equal weights two separate audio channels: (i) the “bird channel” which includes bird songs, and (ii) the “background channel” which combines ambient sound, anthropophony, geophony and insect sounds (Figure 2).

**Figure 3:**
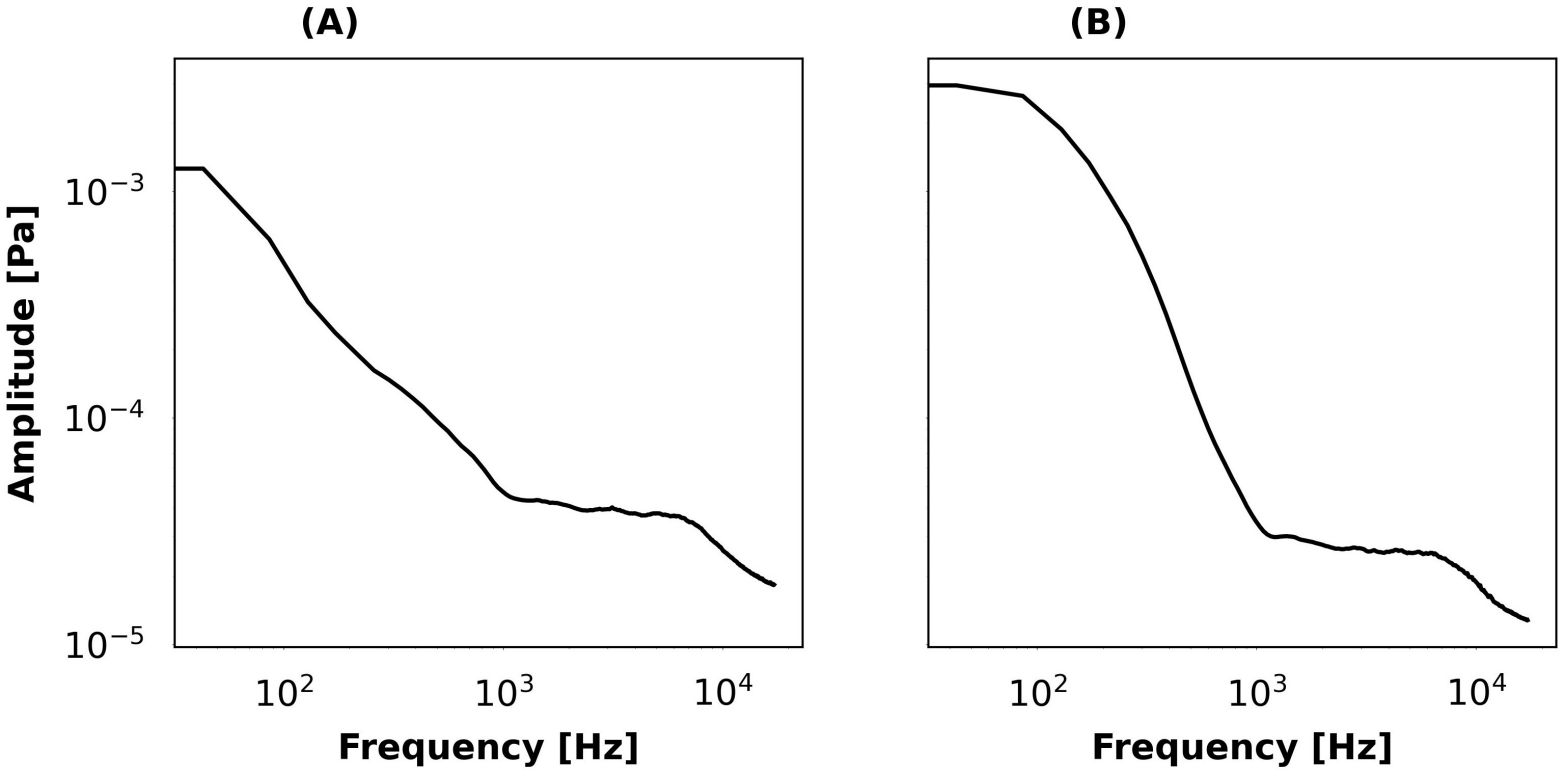
Average long-term power spectrum of the two background sounds recorded in the Risoux forest and used with Evascape: **(A)** ambient sound; **(B)** aircraft noise.

A database of 4,400 assembled soundscapes was generated to simulate four main systematic soundscape manipulations, the effects of which were tested perceptually in psychoacoustical experiments:

- Acoustic biodiversity: eight bird species were selected from the Risoux forest's most prevalent species according to a local inventory (Joveniaux and Chevillard, 2014): *Erithacus rubecula* (European Robin), *Fringilla coelebs* (Common Chaffinch), *Periparus ater* (Coal Tit), *Phylloscopus collybita* (Common Chiffchaff), *Regulus regulus* (Goldcrest), *Sylvia atricapilla* (Eurasian Blackcap), *Turdus merula* (Common Blackbird), and *Turdus philomelos* (Song Thrush). For each species, five audio recordings corresponding to different individuals were selected and extracted from the MNHN online sound library (https://sonotheque.mnhn.fr). The nature (abundance or richness) and size of the simulated bird chorus determined the primary audio-channel of the soundscape assembler. Levels of species abundance ranged from one to five individuals, with all individuals belonging to a single species. Levels of species richness also ranged from one to five individuals, but each individual belonging to a different species. All bird songs were limited to a 5–10 kHz frequency band to avoid the addition of possible residual background sound.
- Background type: background sounds were conveyed via the secondary audio channel of the soundscape assembler. Here, the background represented ambient sound or anthropogenic sounds. An “ambient sound” based on *in-situ* recordings was chosen as the default background type. The ambient sound, which was low-pass in shape and covered the 0–1 kHz frequency range with a cutoff at 100 Hz, was present in most conditions (Figure 3A). “Aircraft” sounds had a low-pass shape and cover the 0–1 kHz range with a cutoff around 300 Hz with a steeper roll-off than the ambient sound (Figure 3B). Although the aircraft noise was extracted from the “strong” aircraft noise category of the Evascape software, the selection made in this study produced a batch of “low/moderately-low” aircraft noise samples, where the average level is 30 dB SPL(A) and never exceeds 40 dB SPL(A).
- Singing behavior: the interactive singing behavior of birds and more precisely, their tendency to avoid song temporal overlap, was controlled for by using a self-organizing, asymmetric rule inspired by the “DESYNC” algorithm (Degesys et al., 2007; Suzuki et al., 2012). This was motivated by the observation that some bird species try to sing during the “silent valleys” of other birds (Brumm, 2006). Here, a “deaf” singing behavior means that simulated birds sing independently from each other, as in the Evascape scenario of a single bird singing. The Evascape Temporal Overlap Avoidance (TOA) algorithm was based on the application of the following four rules: (R1) when there is no song overlap, the bird's next Inter-Song Time Interval (ISTI) is randomly chosen in the observed ISTI distribution of the species; (R2) if there is overlap with more than one bird, the bird that would be heard louder (including attenuation effects due to distance disparities) is considered as the reference bird to which the overlapped bird will change his behavior, introducing asymmetry in these singing interactions (Suzuki et al., 2012); (R3) in the case of song overlap, the overlapped bird next ISTI is calculated so its next song occurs in-between the end of the current overlapping bird song and the beginning of his predicted next song based on the mean of its species' observed ISTI distribution; (R4) if the calculated ISTI from R3 exceeds the maximum ISTI found in the species' observed ISTI distribution, the next ISTI will follow R1.

The duration of each stimulus was set to 5 s because:

- of the fast decay of the auditory trace in echoic and short-term memory for humans (cf. Cowan, 1984; Demany and Semal, 2008);
- of the potential impact of fluctuations on attention and vigilance for each participant;
- psychoacoustic studies conducted with sequences of pure tones indicate that, in the absence of sudden changes in the properties of the tonal sequence, the tendency for stream segregation builds up rapidly over 10 s and then continues to build up more slowly up to 60 s (Moore and Gockel, 2012). Therefore, a 5 s duration may have been too short to allow for this build up of streaming to occur in our participants.

The current target stimuli–that is, bird songs–were acoustically more complex than sequences of alternating tones and the exact time course of this build up is unknown for this class of stimuli showing a rich spectro-temporal structure. Despite the short duration of 5 s, Evascape was designed to guarantee that at least one bird vocalization of each bird would be included in its entirety in the generated stimuli.

### 2.3 Presentation levels

The average Sound-Pressure-Level (SPL) of background sounds and bird chorus assemblages was estimated based on the gain of on-site recorders (Table 1). The higher-than-expected level difference between a single bird and a chorus of 5 birds is due to the attenuation process applied by Evascape, which randomly lowers down bird songs levels and makes the average level of each chorus difficult to predict.

**Table 1:** Average levels of background sounds and bird choruses in dB(Z) (unweighted Sound-Pressure-Level) and dB(A) (A-weighted LAeq values).

**Stimulus**	**Ambient sound**	**Ambient sound min**	**Ambient sound max**	**Aircraft noise**	**Aircraft noise min**	**Aircraft noise max**	**Chorus: 1 individual**	**Chorus: 5 individuals from the same species**	**Chorus: 5 individuals from different species**
dB (Z)	38	24	36	44	29	50	27	44	37
dB (A)	23	18	21	30	19	40	28	40	38

“min” and “max” values correspond respectively to the lowest and highest average value of each class of background sound. Background dB(Z) values are higher than dB(A) values because they show more energy at low audio frequencies [0–1 kHz]. Conversely, chorus dB(Z) values are lower than dB(A) values because they show more energy at high audio frequencies [1–10 kHz].

The level of bird songs was adjusted according to the species average singing peak SPL at 1 m: *Erithacus rubecula* (90 dB SPL)*, Fringilla coelebs* (86 dB SPL), *Periparus ater* (78 dB SPL), *Phylloscopus collybita* (80 dB SPL), *Regulus regulus* (75 dB SPL), *Sylvia atricapilla* (88 dB SPL), *Turdus merula* (87 dB SPL), and *Turdus philomelos* (100 dB SPL). The level of bird songs also varied according to the distance-dependent attenuation function applied by the soundscape assembler. When birds were simulated 50 m from the listener, this resulted in an average attenuation of 25 dB. Thus, the bird-to-background ratio could vary from −8 dB (in the case of *Sylvia atricapilla* propagated at 50 m) to +32 dB (in the case of *Turdus philomelos* propagated at 10 m) for the ambient sound background, and from −10 dB to +30 dB for the aircraft noise background.

**Figure 4:**
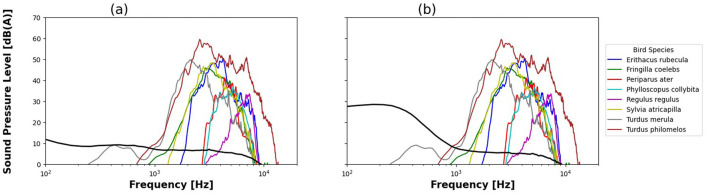
Average Sound Pressure Level spectrum for ambient sound **(a)**, aircraft noise **(b)** and bird species, given in A-weighted dB(A).

The power spectra for the current ambient sound (Figure 4a), aircraft noise (Figure 4b) and bird vocalizations overlap in the 1–10 kHz range. The level of ambient sound and aircraft noise in this frequency interval was low compared to bird levels (ambient sound and aircraft noise levels being about 40 dB SPL lower than bird vocalizations).

### 2.4 Assessment of within- and between-species auditory disparities

Within- and between-species auditory disparities in the spectral and temporal domain were assessed by processing each original bird vocalization throughout a computational model of human auditory system (Thoret et al., 2020; Apoux et al., 2023; Lorenzi et al., 2023). First, the average amplitude-modulation power spectra (AM spectra) were calculated for all the original acoustic samples of each species. Each signal was decomposed by a series of linear gammatone filters tuned in the audio domain between 70 and 11,025 Hz, in order to mimic cochlear frequency analysis (bandwidth = 1 equivalent rectangular bandwidth, ERB). The temporal envelope (AM) was then extracted by taking the module of the Hilbert analytic signal from each narrow-band signal, and decomposed by a second series of so-called modulation filters, tuned to AM rate (bandwidth = 1 octave; Quality factor, Q=1; tuning rates between 0.5 and 200 Hz). Modulation power was calculated at the output of each modulation channel. The AM spectra were finally averaged over all the samples for a given species. Each AM spectrum therefore shows the distribution of AM power as a function of audio frequency (y-axis, frequency, in Hz) and by temporal-modulation rate (x-axis: AM rate, in Hz).

Auditory similarities within and between species were then calculated by computing the distance (i.e., the inner product) between these AM spectra. High values mean high similarity between auditory representations. Similarities across auditory representations computed this way were generally high, indicating that bird vocalizations of the present temperate European biome are relatively close in the spectral and temporal domains and potentially hard to distinguish for human listeners (Table 2). Still, consistent with our initial expectations, lowest similarities were found when comparing these perceptually-relevant representations of bird vocalizations between species rather than within species, suggesting that streaming should be more efficient for assemblages made of individual vocalizations from different species rather than from a single species.

**Table 2:** Auditory similarity within and between species as calculated by a computational auditory model simulating cochlear filtering followed by temporal-envelope decomposition via a modulation filterbank.

**Species**	***Erithacus rubecula***	***Fringilla coelebs***	***Periparus ater***	***Phylloscopus collybita***	***Regulus regulus***	***Sylvia atricapilla***	***Turdus merula***	***Turdus philomelos***
*Erithacus rubecula*	0.96	0.94	0.92	0.90	0.73	0.93	0.72	0.87
*Fringilla coelebs*		0.98	0.83	0.83	0.70	0.97	0.87	0.89
*Periparus ater*			0.97	0.95	0.80	0.86	0.58	0.78
*Phylloscopus collybita*				0.95	0.81	0.83	0.53	0.76
*Regulus regulus*					0.90	0.64	0.37	0.63
*Sylvia atricapilla*						0.98	0.86	0.90
*Turdus merula*							0.91	0.79
*Turdus philomelos*								0.84

Auditory similarity was estimated by the dot product of model outputs to pairs of birds' vocalizations. The higher the value of the dot product, the more similar birds' vocalizations. Within-species similarities are shown in the secondary diagonal (bold characters); between-species similarities are shown by the remaining number in the columns (excluding the secondary diagonal).

### 2.5 Single-judgment numerosity task

Here, a single-judgment method was used to limit contribution of response biases typically associated with numerosity tasks (Warren, 1970; Krueger, 1982). Knowing that single-judgment methods typically require a large number of participants, all experiments were conducted online on the Prolific platform (https://www.prolific.com/). Each participant was asked the following question: “How many birds do you hear in the present recording?” Since the goal of the task was to estimate the number of singing birds and not the total number of bird vocalizations, participants were also instructed that a single bird could sing multiple times. Stimuli contained either individuals from the same species or from different species. For this reason, participants were tested for their capacity to assess biodiversity (either species abundance or species richness) without having to understand the concept of biodiversity. The numerosity task is reminiscent of the one used by Zhong and Yost (2017) for mixtures of speech sounds, except that the present paradigm aimed at minimizing response biases by adopting a single-judgment method.

### 2.6 Participants

One thousand and fifty-one participants were recruited using the prolific online platform. Three hundred seven participants did not pass the headphone screening (see Section 2.7), which left 744 participants who completed the numerosity experiment and a brief individual survey. The species richness condition with ambient background noise and behavior had 128 participants (age = 39.1 ± 13.8 years (mean ± SD), female = 76, male = 52). The richness condition with ambient background noise and no behavior had 118 participants (age = 35.5 ± 12.8 years, female = 58, male = 60). The richness condition with aircraft noise had 125 participants (age = 35.9 ± 14.2 years, female = 59, male = 65, not reported = 1). The abundance condition with ambient noise had 125 participants (age = 34.5 ± 12.6 years, female = 67, male = 58). The richness condition with no background noise had 126 participants (age = 35.9 ± 12.8 years, female = 60, male = 63, not reported = 3). The abundance condition with no background noise had 122 participants (age = 37.8 ± 13.1 years, female = 78, male = 44). All participants gave informed consent by selecting a “continue” button to indicate that they read the consent form and agreed to participate in the experiment. Participants were compensated after completion of the experiment. The experiment procedure was approved by the INSERM ethics evaluation committee (CEEI).

### 2.7 Procedure

All participants completed the online experiment using a personal computer and were asked to wear headphones to perform the task. They first gave their consent to participate in the experiment and were given a brief overview of the task. Next, participants heard a calibration noise stimulus and asked to set their headphone volume to a comfortable listening loudness. The calibration stimulus was a 5 s, pink noise that had a root-mean-squared level of +6 dB relative to the average trial stimulus level. The calibration stimulus level was chosen to ensure the stimulus was audible.

The experiment was split into two sections. In the first section, participants completed a headphones screening task to ensure they were wearing headphones (Woods et al., 2017). The headphone screening task consisted of six trials, and on each trial, participants performed a 3-AFC (alternative forced choice) task where they were asked to identify which of the sound intervals was the quietest. Verifying the use of headphones provided consistent sound presentation across participants and helped standardize the overall listening conditions. If participants passed this screening, they could continue to the second experiment section. The second section consisted of a brief survey to gather information about the headphone type (e.g., headphones vs. earphones), self-reported hearing status, expertise with bird species and ornithology, living area (e.g., urban or rural), and how much time participants spent in natural areas. After the brief survey, participants completed the single-judgment numerosity task. The participants heard a single 5 s sound interval generated using the Evascape software. Participants could only listen to the sound interval once. The participant then entered the number of birds they heard and submitted their results. Participants received a nominal payment for starting the study and a second bonus payment if they successfully completed the headphone screening, survey and numerosity task.

## 3 Results

### 3.1 Biodiversity perception in the absence of background sounds

**Figure 5:**
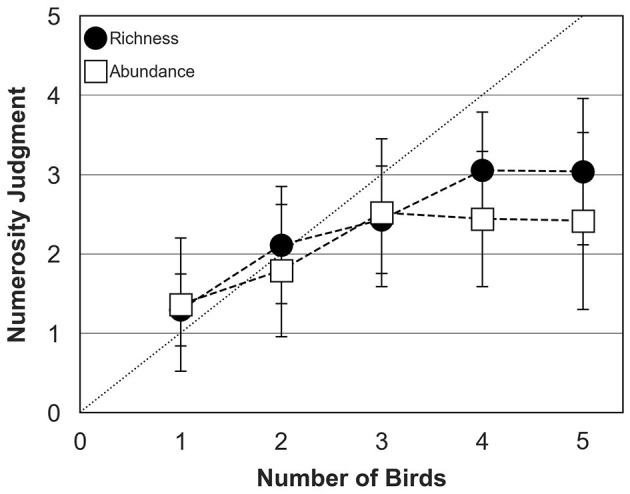
Numerosity judgments of human participants (mean and standard deviation around the mean) for abundance (open squares) and richness (filled circles) conditions in the absence of background sound. The dotted diagonal line represents correct (ideal) responses. In both conditions, bird behavior was controlled by the TOA algorithm.

Figure 5 shows mean numerosity judgments across participants as a function of the actual number of birds composing the chorus (the actual “chorus size”) for the abundance and richness conditions. These preliminary results were obtained in the absence of any background sound but with bird behavior controlled by the TOA algorithm. Numerosity judgments were relatively similar in the abundance and richness conditions when the actual number of birds increased from 1 to 3 birds. In both conditions, participants tended to slightly overestimate the number of birds when a single bird was presented, an effect that likely reflects participant's decision biases with the current single-interval, single-trial paradigm. Participants estimated accurately the number of birds when 2 birds were presented, but systematically under-estimated when bird number exceeded 2, in both conditions. In the abundance condition, numerosity judgments reached an asymptote for choruses of 3 birds with an average judgment of 2.5 birds. In the richness condition, numerosity judgments reached an asymptote for choruses of 4 birds with an average judgment of 3 birds.

### 3.2 Biodiversity perception in the presence of ambient sound

**Figure 6:**
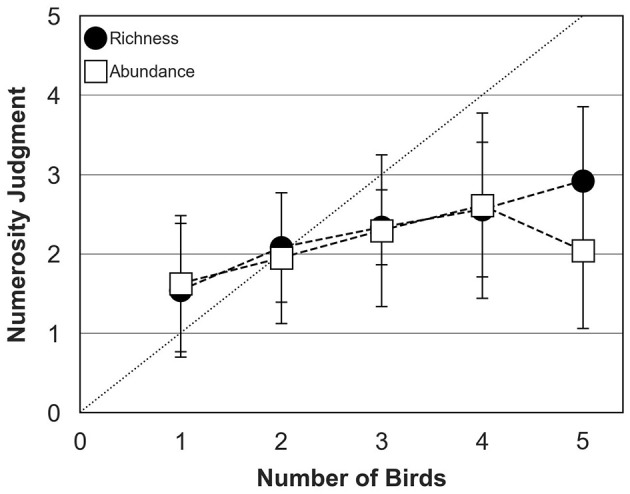
Numerosity judgments of human participants (mean and standard deviation around the mean) for abundance (open squares) and richness (filled circles) conditions in the presence of ambient sound. The dotted diagonal line represents correct (ideal) responses. In both conditions, bird behavior was controlled by the TOA algorithm.

Figure 6 shows mean numerosity judgments across participants measured in the abundance and richness conditions for bird choruses assembled with ambient sound. Bird behavior was controlled by the TOA algorithm in both conditions. As for the preceding experiment (Figure 5), participants tended to slightly over-estimate the number of birds when a single bird was presented but they estimated accurately the number of birds when two birds were presented. In contrast with the preceding experiment where bird choruses were presented without any background sound, numerosity judgments measured in the presence of ambient sound were relatively similar in the abundance and richness conditions when the actual chorus size increased up to 4 birds. In both conditions, participants systematically under-estimated chorus size composed of more than 2 birds, with an average judgment of 2 birds in the abundance condition and 3 birds in the richness condition when 5 birds were presented.

### 3.3 Biodiversity perception in the presence of aircraft noise

**Figure 7:**
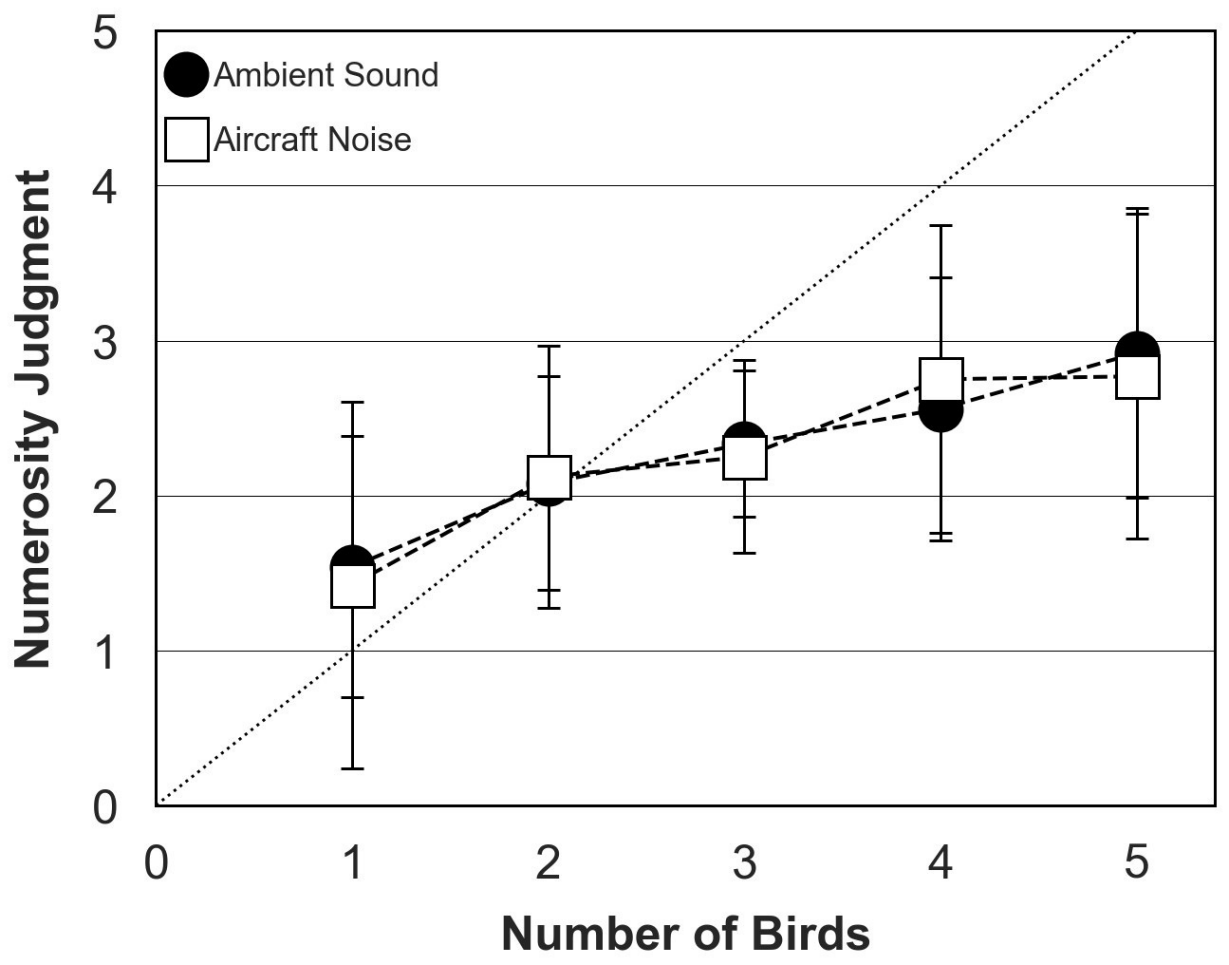
Numerosity judgments of human participants (mean and standard deviation about the mean) for ambient sound (filled circles) and aircraft noise (open squares) conditions. The dotted diagonal line represents correct (ideal) responses. In both conditions, data were collected for the richness condition and bird behavior was controlled by the TOA algorithm.

Figure 7 shows mean numerosity judgments across participants measured for bird choruses assembled with ambient sound or aircraft noise. The data were collected in the richness condition only, with bird behavior controlled by the TOA algorithm. Numerosity judgments were quite similar in both conditions and increased steadily when chorus size increased from 1 to 5 birds. Again, participants systematically under-estimated number of birds for choruses composed of more than 2 birds, with an average judgment of 3 birds in both conditions when 5 birds were presented.

### 3.4 Effect of the behavioral algorithm on biodiversity perception

**Figure 8:**
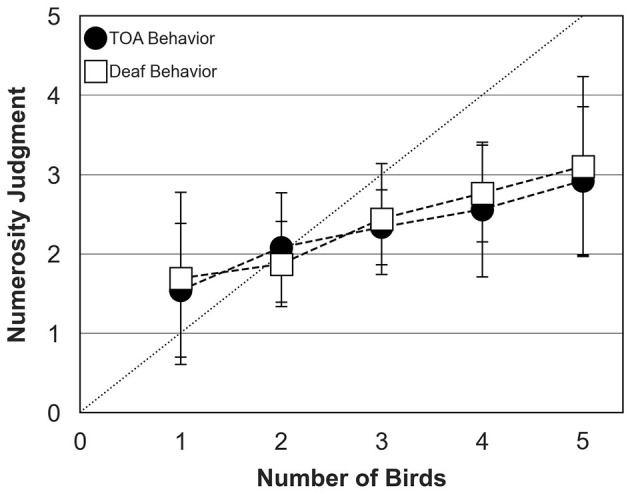
Numerosity judgments of human participants (mean and standard deviation around the mean) for bird behavior controlled by the Temporal Avoidance Algorithm, TOA (filled circles) and the “deaf” behavior algorithm (open squares). The dotted diagonal line represents correct (ideal) responses. In both conditions, data were collected for the richness condition and bird choruses were assembled with ambient sound.

Figure 8 shows mean numerosity judgments across participants, with bird behavior controlled by the TOA algorithm or the so-called “deaf behavior”. The data were collected in the richness condition only, for bird choruses assembled with ambient sound. Numerosity judgments were similar for both types of bird behavior and increased steadily from 1 to 5 birds. Again, participants systematically under-estimated number of birds for choruses composed of more than 2 birds, with an average judgment of 3 birds in both conditions when 5 birds were presented.

### 3.5 Statistical analyses

Two analyses of variance (ANOVA) were conducted on numerosity judgments. The validity of both ANOVAs was checked with diagnostic plots (normality and homoscedasticity of model residuals).

The first ANOVA was conducted to test the effects of bird number (five levels: 1–5), biodiversity type (two levels: individual abundance and species richness) and background sound (two levels: no background and ambient sound). The main effect of bird number was significant [*F*_(4, 498)_ = 32.23; *p* < 0.05], confirming that numerosity judgments were affected by the actual number of birds composing the choruses. There was also a significant interaction between bird number and biodiversity type [*F*_(5, 497)_ = 4.49; *p* < 0.05], consistent with the idea that acoustic disparities within and between species influenced the perceived chorus size. However, the interaction between bird number and background sound [*F*_(5, 497)_ = 1.22; *p* = 0.298] and the three-way interaction between bird number, biodiversity type and background sound were not significant [*F*_(5, 497)_ = 0.89; *p* = 0.487]. *Post hoc* comparisons considering inflation type I risk error (Tukey HSD) indicated that numerosity judgments with 4 or 5 birds were significantly higher than numerosity judgments with 1 or 2 birds (all *p* < 0.05). However, differences in judgment between 3, 4 and 5 birds were not significant (all *p* > 0.05), except for the richness condition without any background, where the difference in numerosity judgments between 3 and 4 birds was significant (*p* < 0.05).

The second ANOVA was conducted to test the effects of bird number (five levels: 1–5), behavior (two levels: TOA and “deaf” algorithm) and background sound (two levels: ambient sound and aircraft noise). Again, the main effect of bird number was significant [*F*_(4, 367)_ = 27.50; *p* < 0.0001]. However, the interaction between bird number and background sound [*F*_(5, 241)_ = 0.25; *p* = 0.94] and the interaction between bird number and behavior were not significant [*F*_(5, 248)_ = 0.47; *p* = 0.80], consistent with the idea that active song interlacing (as implemented by Evascape) does not result in any release from energetic and modulation masking.

### 3.6 Influence of participants' background

**Figure 9:**
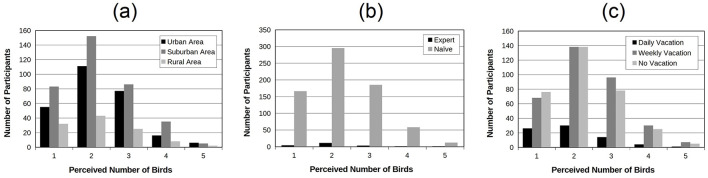
Number of participants as a function of the number of perceived birds. The data were aggregated across all experiments. **(a)** distinguishes participants according to their place of residence (urban, suburban vs. rural areas); **(b)** distinguishes them according to their degree of expertise with birds (ornithologists vs. naive participants) and **(c)** according to the amount of time spent in nature (daily, weekly vs. no vacation). These distributions are quite similar in shape.

In order to address the potential influence of participants backgrounds on the results, three independency Chi-square tests were successively applied on the data. For each test, the independency between the perceived number of birds (from 1 to 5) was tested with, respectively, the place of residence (three levels: urban area, suburban area and rural area; Figure 9a), the degree of expertise (two levels: expert and naïve; Figure 9b) and the time spent in nature (three levels: daily vacation, weekly vacation and no vacation; Figure 9c). All tests were non significant revealing therefore that the auditory ability to estimate the number of birds composing the soundscape does not depend on place of residence, ornithological expertise and time of exposure to natural sounds.

## 4 Discussion

### 4.1 Inefficient auditory perception of biodiversity by human listeners

The present study aimed at assessing the extent to which human listeners are capable of assessing the size of bird choruses. The results reveal that human beings do not accurately estimate bird acoustic biodiversity of natural soundscapes. Indeed, participants slightly over-estimated bird chorus size when a single individual was singing; they accurately estimated bird chorus size when the latter was composed of two individuals, but they systematically under-estimated chorus size for choruses of more than two individuals. It is reasonable to assume that participants' responses have been influenced by decision biases inherent to the present single-interval, single trial psychophysical paradigm. Nevertheless, the current results reveal that the perceived bird chorus size tends to increase systematically with the actual number of individuals in a number of ecologically-valid conditions, demonstrating some form of auditory sensitivity to biodiversity that should generalize to real natural settings. Indeed, the soundscape assembler used to generate the acoustic stimuli presented to our participants was designed to take into account critical biological, geophysical and acoustic factors known to shape natural soundscapes (cf. Grinfeder et al., 2022a) such as those associated with a cold temperate forest. Still, it is important to note that the soundscape assembler did not provide spatial information as to the position (azimuth and elevation) of birds. Had this been the case, the perceived size of the bird chorus would most likely have been larger, given related work conducted with mixtures of human voices (e.g., Kawashima and Sato, 2015; Zhong and Yost, 2017).

Although sub-optimal, the auditory sensitivity of humans to biodiversity may still be of use to assess the global health of a given habitat for human listeners, and play the role of an alarm system when an environment has poorer-than-expected biodiversity. In any case, the current findings suggest that the size of natural auditory scenes is typically small, not larger than three bird vocalizations, consistent with the outcome of a similar investigation conducted with human voices, (man-made) environmental sounds and tonal signals (Kawashima and Sato, 2015; Weller et al., 2016; Vitevitch and Siew, 2017; Zhong and Yost, 2017; Roberts et al., 2019; for a review see Kwak and Han, 2020). This finding warrants further work to assess the origin of the observed response biases, and develop novel paradigms (e.g., forced-choice tasks) to measure biodiversity perception while controlling better for these criterion effects.

### 4.2 Relevance of auditory scene analysis principles to natural soundscape perception

Our study also aimed at assessing the extent to which general principles known to control for auditory scene analysis in humans (Bregman, 1990; Moore and Gockel, 2012) apply to natural scenes such as those produced by a cold forest in Europe. The results showed that potentially audible acoustic disparities across bird vocalizations – as crudely estimated by a computational auditory model – influence significantly the size of the auditory scene, as estimated by the number of birds perceived by human observers. More precisely, between-species spectro-temporal similarities across bird vocalizations were found to significantly – although modestly – enhance numerosity judgments by human listeners, consistent with an involvement of auditory grouping mechanisms. Further work should investigate the influence of additional similarities on the perceived chorus size, such as differences in level across bird vocalizations (as produced by distance to the observer or by between-species differences).

### 4.3 Robustness of biodiversity perception for natural scenes

Our results indicate that biodiversity estimates, both in terms of abundance and richness, were only slightly affected by the presence of ambient background noise. This suggests that ambient sound, understood as the undistinguishable mixture of biophony, geophony, and anthropophony, can be used as “baseline” background for subsequent experiments, enhancing the ecological validity of the stimulus.

Our study also showed that anthropogenic sounds (here, aircraft noise) do not affect the perceived size of bird choruses, for aircraft noise levels considered in the present study. Not surprisingly, aircraft noise level was higher than ambient sound (by about 20 dB) at very low audio frequencies (<0.5 kHz) only. Although minimal, aircraft noise should yield more “upward spread of masking” (i.e., higher growth rate of masking for maskers lower in audio frequency than the target signal, compared to maskers at the target signal audio frequency; Egan and Hake, 1950) than ambient noise, and alter slightly bird sound audibility in human participants, at least for those bird species showing low (around 1 kHz) audio frequency components. Still, these energetic masking effects resulting from the asymmetry of cochlear filters for humans (Moore and Gockel, 2012) should be rather modest. Aircraft noise could also yield modulation masking and modulation detection interference effects susceptible to alter auditory discrimination of bird vocalizations. These two forms of within- and across-channel non-energetic masking (Houtgast, 1989; Yost et al., 1989) result from the slow amplitude modulations conveyed by aircraft noise (Lincke and Pieren, 2023).

These findings suggest that auditory mechanisms engaged in biodiversity assessment are relatively robust to masking and interference. Still, studies investigating the effects of aircraft noise on hearing and health (Basner et al., 2017) suggest that masking effects may alter the detectability of bird vocalizations if propagated further away. This also does not preclude the negative effects of aircraft and other machine noise on human listening experience in nature (Buxton et al., 2021). Further work is therefore warranted to explore the effects of aircraft noise in other naturalistic conditions, in particular by varying the signal-to-background level.

### 4.4 Animal singing behavior does not benefit natural soundscape perception

Our dedicated algorithm allowed us to test for a role of masking release and glimpsing mechanisms in biodiversity perception, as found previously for speech production and perception in non-stationary, single or multi-talker backgrounds (see Cooke and Lu, 2010). The results showed that aspects of bird behavior, namely temporal avoidance between bird vocalizations, did not affect numerosity judgments, inconsistent with the idea that bird behavior promotes release from energetic and modulation masking.

### 4.5 Conclusions

In conclusion, this pilot study indicates for the first time that human observers possess the capacity to estimate – even grossly – changes in bird biodiversity embedded in natural soundscapes, an auditory attribute that may be diagnostic of the quality of the close environment and play a role in the autonomic response of the human nervous system to natural sounds (Buxton et al., 2021). The present results, although preliminary, suggest that this capacity is constrained by general auditory mechanisms already demonstrated for communication and urban settings (see Kawashima and Sato, 2015; Weller et al., 2016; Zhong and Yost, 2017). It is reasonable to assume that this auditory capacity predates previously observed capacities for the perception of “speech cocktails” in urban situations, which appeared more recently in human history. Further work is warranted to explore this capacity for natural environments auditory analysis, by simulating more accurately ecological factors at work in ecosystems such as spatial factors (e.g., distance between birds and receiver, reverberation effects, etc.) and animal behavior, assessing the potential impact of man-made acoustic pollutants, and more broadly, unraveling the role of biodiversity perception in humans sensory, cognitive and emotional processing of natural sound.

## Data Availability

The raw data supporting the conclusions of this article will be made available by the authors, without undue reservation.
